# COVID-19 pandemic and unmet need for family planning in Nigeria

**DOI:** 10.11604/pamj.2021.40.186.27656

**Published:** 2021-11-26

**Authors:** Turnwait Otu Michael, Richard Dele Agbana, Tolulope Funmilola Ojo, Olasumbo Bilikisu Kukoyi, Alfred Stephen Ekpenyong, Damian Ukwandu

**Affiliations:** 1 Demography and Population Studies, Sociology, University of Ibadan, Ibadan, Nigeria,; 2 Department of Public Health, Afe Babalola University, Ado-Ekiti, Nigeria,; 3 Department of Sociology, Niger Delta University, Amassama, Nigeria,; 4 Department of Public Management and Governance, University of Johannesburg, Johannesburg, South Africa

**Keywords:** COVID-19, unmet need for family planning, contraceptive methods, pandemic lockdown, Nigeria

## Abstract

**Introduction:**

the unmet need for family planning is a global health burden. The lockdown occasioned by the COVID-19 pandemic has reduced access to contraceptives, especially in the developing countries. This study examined the predictors of the unmet need for family planning during the COVID-19 pandemic lockdown in Nigeria.

**Methods:**

the study adopted a cross-sectional analytical survey design. A self-designed questionnaire was administered to 1,404 adult respondents aged 18 years and above. The data was generated through the use of online Google survey and analyzed with SPSS version 25. The results were presented using descriptive and logistic regression at p≤0.05.

**Results:**

fourty-seven percent of the respondents were females and 58.8% were married. The four major reasons for non-access to contraceptive methods during the lockdown were: fear of visiting health facility (77.9%), locked drug/chemist stores (51.2%), the restriction of movement (47.6%) and a lack of access to health care providers (42.9%). Predictors of unmet need for family planning were: aged 26-33 (OR = 1.912, 95% CI: 1.02-3.55), married/cohabiters (OR = 3.693, 95% CI: 2.44-5.58), tertiary education (OR = 0.272, 95% CI: 0.13-0.54), Yoruba ethnicity (OR=1.642, 95% CI: 1.02-2.62), rural residence (OR = 0.554, 95% CI: 0.36-0.85) and 2-4 children born (OR = 3.873, 95% CI: 2.32-6.45).

**Conclusion:**

a significant proportion of Nigerians experienced an unmet need for family planning during the COVID-19 lockdown. Prioritizing the access to contraceptives during the pandemic would not only allow women and men to correctly plan childbirth, it also reduces maternal risks, poverty and undesirable fertility rates.

## Introduction

COVID-19, which is also known as the coronavirus disease has held the world in a vice grip for several months now. The World Health Organization declared the disease a global pandemic on 11^th^ March 2019 after recording 118,000 cases and 4,291 deaths in 114 countries [1]. COVID-19 has disrupted all spheres of human existence: health, economy, politics, education and even the family unit [2-4]. It has increased the burden of the unmet need for family planning, thus making individuals, families and nations to encounter challenges in accessing modern contraceptives [5,6]. Modern contraceptive methods for family planning include the male and female condoms, intrauterine device (IUD), injectables, implants, pills and emergency contraception, while the traditional methods include rhythm, withdrawal, local herbs and concoctions [7,8].

The barriers to accessing family planning methods during the COVID-19 pandemic range from the fear of contacting the virus at health facilities, the restriction of movement, the closure of borders, the shutting down of contraceptive manufacturing factories, to the closure of some healthcare facilities [9-11]. The COVID-19 pandemic has made governments and managements of health facilities in many countries to focus most of the available human, material and financial resources to the fight against coronavirus with little or no attention given to the production, distribution and administration of family planning commodities/services [12,13]. Consequently, family planning services across the globe has become less available and inaccessible for many people. This is in spite of the fact that family planning/reproductive health care is among the essential services that must be made available to everyone during the pandemic [13,14].

The unmet need for family planning refers to the intention to stop or delay childbearing without using or accessing a contraceptive method [7,15]. It has accounted for the high population rates, increased rates of unwanted pregnancies, increased abortion and uncontrolled fertility rates, especially in the developing countries [16,17]. The lack of access to modern contraceptives which was occasioned by the COVID-19 pandemic has further exposed women/girls and their newborn babies to avoidable maternal and infant morbidity and mortality [18,19]. The United Nations Population Fund (UNFPA), particularly, estimated that about 47 million women in 114 low- and middle-income nations, including Nigeria, would lack access to modern contraceptives if the COVID-19 lockdown continues for another six months [20]. The World Health Organization advocates for a balance between the fight against COVID-19 and the unmet need for sexual and reproductive health so that health outcomes are positive [21].

While the COVID-19 lockdown and restriction of movement kept some spouses/partners apart, it forced others to spend more time together [22,23]. Consequently, they engage in more sexual activity than they did before the lockdown [23-25]. When fertile, partners constantly have sexual relations without contraceptives, the result is a lot of unplanned pregnancies [26,27]. This situation could lead to an unchecked population explosion, especially in Nigeria, where there is already a teeming population of over 208 million people [28]. It is worthy of note that although Nigeria´s population is not just the highest in Africa, the country is the fourth most fertile nation in Africa, and is currently the seventh most populous nation in the world, with a total fertility rate (TFR) of 5.3 [7]. This rate is above the global and continental average rates of 2.3 and 4.4 respectively [7,29].

Nigeria also has 48% unmet need for family planning among sexually active unmarried women and 19% among currently married women [7]. Due to the country´s high fertility rates and intentions [30,31], the outbreak of COVID-19 is likely to hamper the commitment to the 2020 family planning goals [29]. As such, efforts targeted at controlling the fertility rate and improving reproductive health care, especially during COVID-19 pandemic will also adversely be affected. In light of these observations, the study examines the influence of COVID-19 pandemic on contraceptive use/family planning in Nigeria.

## Methods

**Study design and setting:** the study adopted a cross-sectional analytical survey design. The fieldwork was conducted around 24^th^ May to 30^th^ July 2020, during the COVID-19 lockdown in Nigeria. The respondents were an adult population of age 18 years and above which cut across the six geopolitical zones of Nigeria. The country, Nigeria, which has the largest population in Africa, is located in the West Africa region.

**Sample:** the sample size for this study was determined by Cochran´s [32] formula:


$$n_{0}=\frac{z^{2}P\left(1-P\right)}{e^{2}}$$

Where: n_o_= sample size; Z = statistics level of confidence (1.96); P = expected prevalence or proportion of child fosterage in the study area (0.50); 1 = constant; e = marginal error (0.05). no = 1.962[0.50(1-0.50)]/0.05^2^ = 3.8416[0.50(0.50)]/0.05^2^ = 384 plus 10% (attrition) = 422; no = 422*3.8 = 1,604 (for Nigerian adult population) Adhering to the calculated sample size formula, a total of 1,604 copies of the online questionnaire were expected. However, only 1,404 copies of the questionnaire were retrieved and found valid for this study.

**Instrumentation/data collection:** a self-administered questionnaire titled “COVID-19 and contraceptives/family planning” was the instrument used for data collection. The questionnaire was divided into three sections (A, B and C). Section A - demographic characteristics of respondents, section B - knowledge of COVID-19, and section C - methods of contraceptive/family planning. The items on the instrument were guided by a literature review which includes the World Health Organization´s questions and answers on COVID-19 updates [33] and the demographic and health survey guides on contraceptive/family planning [7]. The questionnaire was designed on Google form and was administered via a link that was sent to respondents´ WhatsApp, Twitter, Facebook and email. There was also a suggestion that respondents should kindly respond to the questionnaire and forward the link to their social media contacts, including friends, colleagues and family members, to help increase the number of respondents. The questionnaire had 32 items, coded with “yes” or “no” responses; yes = 1 and no = 0. Every correct answer received 1 score and incorrect response received 0 score. Others had multiple responses.

**Data analysis:** the responses provided by respondents were stored in the Google form, retrieved from the Google drive, saved in an Microsoft Excel spreadsheet and imported into the SPSS software (IBM version 25), after data cleaning, for further analysis. Missing data were uncommon in the datasets. However, seven submissions with missing variables on unmet need for family planning were dropped from the analysis. The data were analyzed at descriptive (frequencies and percentages) and multivariate (binary logistic regressions) levels. Binary logistic regression explains the prediction of the odds of a case by independent variables to show the probability of variable outcomes. The probability of a binary event in the logistic model used was:


$$\mathrm{Pr}\left(Y=1/X\right)=\exp\left(\beta_{0}+\beta_{1}X\right)/1+\exp\left(\beta_{0}+\beta_{1}X\right)=1/1+\exp\left(-\beta_{0}-\beta_{1}X\right)$$

where (Y=1) is the logarithm of the odds of a response event (in this case, non-access to contraceptive/family planning services), while (Y=0) is a response non-event (access to contraceptive/family planning services). The magnitude of the association of X and Y is represented by the slope β_1_. Given that X is binary, only two values were needed for consideration: X = 0 and X = 1. To account for the odds ratio (OR), the values were combined to give OR_yx_= exp(β_1_) at 0.95 confidence level.

**Ethical considerations:** the ethical approval for this study was obtained from the Afe Babalola University Ado-Ekiti (ABUAD) Ethical Committee (Ref: AB/EC/20/12118). The study adhered to the ethical regulations guiding online surveys. All the participants consented to participate in the study after reading the purpose of the research which was written on the first page of the online Google form (questionnaire). They had to understand this and gave their consent before they were allowed to proceed to the main study´s questions. The respondents were informed that participation in the study was voluntary and that they had the right to skip any question which they felt uncomfortable with. They were made to understand that they could also withdraw entirely from participating in the study at any stage without being penalized. The confidentiality and privacy of respondents were also secured. None of the respondents was asked to include their names or address. The responses received were used solely for the research´s purpose.

## Results

**Background characteristics of study participants:** of the 1,404 respondents that were sampled for the study, 47% were females. Majority of the respondents (73.9%) were aged 26-41 years. More than half of the respondents (58.8%) were married. Majority of the respondents (81.2%) had tertiary education. More than half of the respondents (65.8%) were Christians. Nearly half of the respondents (48.3%) were Yoruba. Majority of the respondents (71.2%) were urban dwellers. Thirty-nine percent of the respondents had no children, 31% had 1-2 children while others (30.6%) had more than 2 children (Table 1).

**Table 1:** background characteristics of respondents

Characteristics	Frequency (N=1,404)	Percentage
**Sex**		
Male	738	52.6
Female	666	47.4
**Age (years)**		
18-25	99	7.1
26-33	603	42.9
34-41	435	31.0
42-49	234	16.7
50+	33	2.4
**Marital status**		
Single	483	34.4
Married	825	58.8
Cohabiters	69	4.9
Divorced/separated	27	1.9
**Education**		
Primary	117	8.3
Secondary	147	10.5
Tertiary	1140	81.2
**Religion**		
Christianity	923	65.8
Islam	462	32.9
Other	18	1.3
**Ethnicity**		
Hausa	247	17.6
Yoruba	678	48.3
Igbo	182	13.0
Other	297	21.2
**Residence**		
Urban	999	71.2
Suburban	177	12.6
Rural	228	16.2
**No. of children born**		
0	540	38.5
1-2	435	31.0
3-4	321	22.9
5 or more	108	7.7

**Figure 1:**
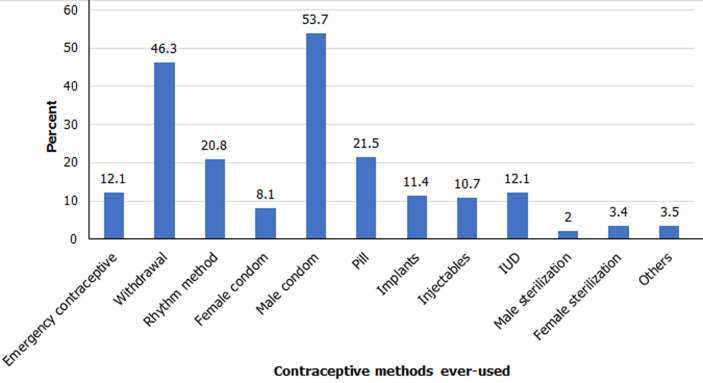
distribution of respondents by contraceptive methods ever-used

**Contraceptive methods ever used:** the distribution of respondents by the contraceptive methods ever used shows that male condoms (53.7%), withdrawal (46.3%), pill (21.5%) and rhythm (20.8%) were the four most commonly mentioned contraceptive methods adopted by the respondents (Figure 1). Among these four methods, condoms and pills are among the modern contraceptive methods while withdrawal and rhythm are traditional methods. These traditional methods of contraceptives are unreliable and expose users to unplanned pregnancy. Having a significant number of the respondents (about 46%) using withdrawal methods means that a lot of them are exposed to unplanned pregnancy and sexually transmitted infections such as HIV/AIDS. The chances are higher when an unfaithful partner is involved.

**Figure 2:**
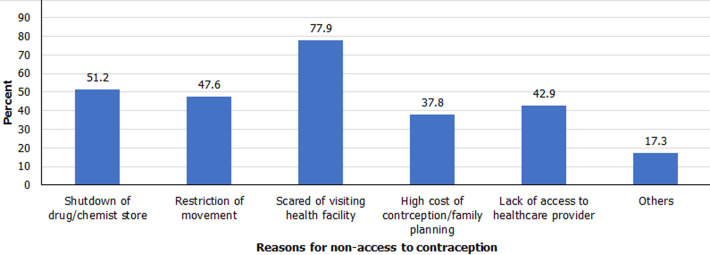
reasons for non-access to contraceptive methods during the COVID-19 lockdown

**Reasons for non-access to contraceptive methods:** the distribution of respondents by reasons for non-access to contraceptive methods during the COVID-19 lockdown shows that the four major reasons for non-access to contraceptive methods during the period were: scared of visiting health facility for fear of contacting coronavirus among other diseases (77.9%), shutdown of drug/chemist stores (51.2%), restriction of movements (47.6%) and lack of access to health care providers (42.9%) (Figure 2). Restricting unnecessary movements to prevent the spread of COVID-19 is appropriate and medically advisable. However, limiting access to contraceptive methods among sexually active individuals during the pandemic has adverse effects. It could increase unplanned pregnancies, and expose women/girls to induced abortion, vicious poverty and maternal risks. This is especially so in a country like Nigeria where induced abortion is illegal and access to health care services is limited.

**Predicators of unmet need for family planning:** in Table 2, we present the odds ratio for the unmet need for family planning logistic regression models by selected characteristics of respondents. The results are presented in three models (model 1, 2 and 3). Model 1 consists of the socio-demographic characteristics of respondents: sex, age, marital status, education and ethnicity. Model 2 presents the explanatory effect of residence and number of children born on the dependent variable (unmet need for family planning). These two variables (residence and number of children born) are separated from predictors included in model 1 because during the COVID-19 lockdown, place of residence (urban, suburban and rural) had strongly determined access to essential commodities, strictness of the lockdown and adherence to COVID-19 precautions. Also, the number of children born is largely documented to influence the desire for a child or more children [26,34]. Model 3 consists of all the independent variables earlier included in models 1 and 2 to check for variations that may occur. The results show that among the seven explanatory measures (predictors) in the models, six variables (age, marital status, education, ethnicity, residence and number of children born) are statistically significant in predicting the unmet need for family planning during the COVID-19 lockdown. Sex shows no statistically significant effect on the dependent variable.

**Table 2:** odds ratio for unmet need for family planning logistic regression models by respondents’ selected characteristics

Characteristics	Model 1			Model 2			Model 3		
	Odds ratio	95% C.I.		Odds ratio	95% C.I.		Odds ratio	95% C.I.	
**Sex**									
Male (RC)	1.000						1.000		
Female	0.816	0.62	1.05				0.856	0.64	1.12
**Age (years)**									
18-25 (RC)	1.000						1.000		
26-33	1.992**	1.11	3.55				1.912*	1.02	3.55
34-41	2.238**	1.19	4.19				1.748	0.87	3.49
42+	1.190	0.61	2.30				0.820	0.37	1.78
**Marital status**									
Single (RC)	1.000						1.000		
Married/cohabiters	6.847***	5.05	9.84				3.693***	2.44	5.58
Divorced/separated	0.852	0.33	2.19				0.638	0.63	0.22
**Education**									
Below tertiary (RC)	1.000						1.000		
Tertiary	0.373***	0.21	0.63				0.272***	0.13	0.54
**Ethnicity**									
Hausa (RC)	1.000						1.000		
Yoruba	2.686***	1.77	4.07				1.642*	1.02	2.62
Igbo	2.119**	1.29	3.46				1.768*	1.04	2.98
Other	2.645***	1.68	4.15				2.164**	1.33	3.52
**Residence**									
Urban (RC)				1.000			1.000		
Suburban				1.274	0.88	1.84	1.479	0.98	2.23
Rural				0.543***	0.38	0.76	0.554**	0.36	0.85
**No. of children born**									
0 (RC)				1.000			1.000		
1-2				7.541***	5.61	9.12	2.911***	1.84	4.58
3-4				7.198***	5.20	9.96	3.873***	2.32	6.45
5+				1.189	0.75	1.87	0.228*	0.22	0.88
Model chi-square	321.347***			327.584***			410.441***		
Nagelkerke R square	0.274			0.279			0.339		
-2 Log likelihood	1608.954			1602.717			1519.860		

Significant at p<0.05*; p<0.01**; p<0.001***; RC = reference category; N = 1,403

In model 1, respondents with ages 26-33 and 34-41 years have 1.9 and 2.2 times higher unmet needs for family planning respectively than those with aged 18-25 years. The married/cohabiters have 6.8 times higher unmet need for family planning than the singles. Those with tertiary education have 0.3 times lower unmet needs for family planning than those with lower education. People from Yoruba, Igbo and other ethnic groups have higher unmet need for family planning than those from the Hausa ethnic group at 2.6, 2.1 and 2.6 times respectively. In model 2, rural dwellers have lower unmet needs for family planning than urban dwellers. Those with 1-2 and 3-4 children have 7.5 and 7.1 times higher unmet needs for family planning respectively than those with no children.

In model 3, with the inclusion of all the seven predictive variables, some variables receive lower statistically significant likelihood while others become insignificant. For instance, while those with aged 26-33 years retained significant effect, those with aged 34-41 did not. The times of likelihood for married/cohabiters relative to single reduced from 4.8 times to 3.6 times after including residence and number of children born in the equation. Similarly, times of likelihood for all ethnic groups relative to people of Hausa descent reduced from 2.6 to 1.6 times for Yorubas, 2.1 to 1.7 times for Igbos and 2.6 to 2.1 times for other ethnic groups. The times of likelihood for number of children born relative to no child reduced from 7.5 to 2.9 times for respondents with 1-2 children and 7.1 to 3.8 times for respondents with 3-4 children.

## Discussion

This study was designed to investigate the influence of the COVID-19 pandemic on the unmet need for family planning in Nigeria. The evidence from the findings shows that COVID-19 pandemic lockdown influences the unmet need for family planning. This finding corroborates previous research which showed that the outbreak of COVID-19 pandemic has disrupted access to family planning services [5,6,12,16]. In order of ranking, the four commonly ever-used contraceptive methods mentioned by the respondents were male condom, withdrawal, pill and rhythm. Among these methods, condoms and pills are classified under modern contraceptive methods while withdrawal and rhythm are grouped under traditional methods [7,8,26]. The traditional methods of contraceptives are unreliable and expose users to unplanned pregnancy.

In order of ranking, the major reasons for non-access to contraceptive methods during the lockdown were: scared of visiting health facility for fear of contacting coronavirus among other diseases, shutdown of drug/chemist stores, restriction of movements and lack of access to health care providers. These findings are congruent with previous studies which state that barriers to accessing family planning methods during the COVID-19 pandemic includes the fear of contacting the virus at health facility, restriction of movement and shut-down of contraceptive manufacturing factories [9,10].

Restricting unnecessary movements to prevent the spread of COVID-19 is appropriate and recommended. However, limiting access to contraceptive methods among sexually active individuals during the COVID-19 pandemic is risky and could increase unplanned pregnancy, induced abortion, poverty and maternal morbidity and mortality among the inhabitants [20,21,35]. Our study´s finding deviates from the research conducted before the COVID-19 pandemic in Nigeria which revealed that the most common reasons for the discontinuation of contraceptives were the desire to get pregnant [7] and the perceived side effect of the method [36,37].

The study´s finding revealed that among the seven explanatory measures included in the models, six variables (age, marital status, education, ethnicity, residence and number of children born) were statistically significant in predicting the unmet need for family planning during the lockdown. Those with ages 26-41 years had a higher unmet need for family planning than others. Previous research showed that Nigeria has 28% sexually unmarried women and 12% married women (aged 15-49 years) currently using modern contraceptives (national population commission (NPC) and inner city fund (ICF), 2019). Having low contraceptive use prior to COVID-19 pandemic and higher unmet need for family planning during lockdown, among sexually active population (aged 26-41 years) may likely expose Nigeria to higher fertility rates that outweigh its available resources and increase poverty among mothers and children [27,34,35,38].

Our finding revealed that married/cohabiters had higher unmet need for family planning than the single persons. This finding deviates from previous research conducted before COVID-19 pandemic which showed that unmet need for family planning was higher among sexually active unmarried women than married women [7,26,39]. The incongruency in findings could be associated with the period of research, and the fact that while past studies included only women of reproductive age, our study includes both sexes. Those with tertiary education have lower unmet need for family planning than others. This finding corroborates previous research that lower unmet need for contraceptive use is recorded among women with more than secondary education [7,40,41], and further indicates that the need for higher education is key to attending met need for family planning. This is because it equally inspires the right decision making towards contraceptive especially in developing countries where access to modern contraceptives is generally low [15,17,42,43].

Our study found that Yoruba, Igbo and other ethnic groups have higher unmet need for family planning than people of the Hausa ethnic group. Rural dwellers were also found to have lower unmet need for family planning than urban dwellers. This is consistent with previous research that contraceptive use and unmet need for family planning are both higher among urban dwellers than rural dwellers [7,36]. Those with 1-4 children have higher unmet need for family planning than those without children. Previous studies reported that women with two or more children have higher contraceptive use than those with only one child [36,40].

The limitations of this study are that the researchers did not separate males from females during the analysis to show the individual gender unmet need for family planning across the socio-demographics of the study´s respondents. An aspect of child spacing in family planning was not measured. Those with non-access to internet were excluded. The data were also gathered via self-report questionnaire. As such the researchers were unable to define a causal relationship. On the other hand, the strengths of the study include a combination of male and female respondents in checking for the effect on unmet need for family planning, reasons for nonuse/discontinuation of contraceptives, and the use of distinct models to separately and jointly examine the effects of the explanatory variables on unmet need for family planning in Nigeria during the COVID-19 lockdown. The use of large datasets also provided the opportunity to generalize this study findings to other countries in sub-Saharan Africa with similar characteristics.

## Conclusion

The COVID-19 pandemic lockdown has disrupted access to contraceptives in Nigeria thus changing the dynamics of the unmet need for family planning. Individuals who initially used a modern method of contraceptive now lack access to their contraceptive method of choice for several reasons including the fear of visiting health care facility, shutdown of drug/chemist stores, restriction of movements and lack of access to health care providers. The factors which significantly influence the unmet need for family planning in the country during the lockdown include age, marital status, education, ethnicity, residence and number of children born by respondents.

### What is known about this topic

- COVID-19 has increased the non-accessibility -and -availability of family planning services across the globe, in spite of the fact that family planning/reproductive health care is an essential health service that must be made available to everyone at all times;
- The unmet need for family planning has accounted for high population rates, increasing unplanned pregnancy, and induced abortion especially in the developing countries.

### What this study adds

- The four major reasons for non-access to contraceptive methods during the period are: scared of visiting health facility for fear of contacting coronavirus among others diseases (77.9%), shutdown of drug/chemist stores (51.2%), restriction of movements (47.6%) and lack of access to health care providers (42.9%);
- Age, marital status, education, ethnicity, residence and number of children born are statistically significant in predicting the unmet need for family planning during the lockdown occasioned by the COVID-19 pandemic; those with ages 26-41 years; the married/cohabiters; and those with 1-4 children have higher unmet need for family planning.
